# Can patients be trained to expect shared decision making in clinical consultations? Feasibility study of a public library program to raise patient awareness

**DOI:** 10.1371/journal.pone.0208449

**Published:** 2018-12-12

**Authors:** Evehouenou Lionel Adisso, Valérie Borde, Marie-Ève Saint-Hilaire, Hubert Robitaille, Patrick Archambault, Johanne Blais, Cynthia Cameron, Michel Cauchon, Richard Fleet, Jean-Simon Létourneau, Michel Labrecque, Julien Quinty, Isabelle Samson, Alexandrine Boucher, Hervé Tchala Vignon Zomahoun, France Légaré

**Affiliations:** 1 Tier 1 Canada Research Chair in Shared Decision Making and Knowledge Translation, Université Laval, Quebec City (QC, Canada); 2 Centre de recherche sur les soins et les services de première ligne de l'Université Laval (CERSPUL), Centre intégré universitaire de santé et de services sociaux (CIUSSS) de la Capitale-Nationale, Quebec City (QC, Canada); 3 Freelance science communication specialist, Quebec City (QC, Canada); 4 Bibliothèque de Québec (Quebec City network of public libraries), Quebec City (QC, Canada); 5 Department of Family Medicine and Emergency Medicine, Université Laval, Quebec City (QC, Canada); 6 Department of Anesthesiology and Critical Care Medicine, Division of Critical Care Medicine, Université Laval, Quebec City (QC, Canada); 7 Centre intégré de santé et services sociaux de Chaudière-Appalaches (site Hôtel-Dieu de Lévis), Lévis (QC, Canada); 8 Centre de recherche du CHU de Québec-Université Laval, Hôpital St-François D’Assise, Quebec city (QC, Canada); 9 Canadian Institutes of Health Research, Quebec City (QC, Canada); 10 Quebec SPOR SUPPORT Unit, Quebec City (QC, Canada); University of Birmingham, UNITED KINGDOM

## Abstract

**Introduction:**

Shared decision making (SDM) is a process whereby decisions are made together by patients and/or families and clinicians. Nevertheless, few patients are aware of its proven benefits. This study investigated the feasibility, acceptability and impact of an intervention to raise public awareness of SDM in public libraries.

**Materials and methods:**

A 1.5 hour interactive workshop to be presented in public libraries was co-designed with Quebec City public library network officials, a science communication specialist and physicians. A clinical topic of maximum reach was chosen: antibiotic overuse in treatment of acute respiratory tract infections. The workshop content was designed and a format, whereby a physician presents the information and the science communication specialist invites questions and participation, was devised. The event was advertised to the general public. An evaluation form was used to collect data on participants’ sociodemographics, feasibility and acceptability components and assess a potential impact of the intervention. Facilitators held a post-workshop focus group to qualitatively assess feasibility, acceptability and impact.

**Results:**

All 10 planned workshops were held. Out of 106 eligible public participants, 89 were included in the analysis. Most participants were women (77.6%), retired (46.1%) and over 45 (59.5%). Over 90% of participants considered the workshop content to be relevant, accessible, and clear. They reported substantial average knowledge gain about antibiotics (2.4, 95% Confidence Interval (CI): 2.0–2.8; *P <* .*001*) and about SDM (4.0, 95% CI: 3.4–4.5; *P <* .*001*). Self-reported knowledge gain about SDM was significantly higher than about antibiotics (4.0 versus 2.4; *P <* .*001*). Knowledge gain did not vary by sociodemographic characteristics. The focus group confirmed feasibility and suggested improvements.

**Conclusions:**

A public library intervention is feasible and effective way to increase public awareness of SDM and could be a new approach to implementing SDM by preparing potential patients to ask for it in the consulting room.

## Introduction

Shared decision making (SDM) is a process whereby decisions are made together by health professionals and their patients and/or families [1]. Together they consider the best scientific evidence about the risks and benefits of all available options, a process that can be supported by the use of decision aids. Their decision takes into account the values and preferences of the patient, which are solicited and acknowledged by the health professional [2]. There is an ethical imperative for health professionals to share important decisions with patients [3] and SDM is appearing in legislation governing healthcare in numerous countries [4].

SDM is associated with several positive health outcomes, one of which is a reduction of overuse of treatments and services [5]. For example, a program to train health professionals in SDM was effective at reducing overuse of antibiotics without harming patients [6]. This is important in a context where antibiotics resistance has become a major public health problem [7]. Finally, by acquiring decision-making competencies and being supported in their decisions, patients can make informed decisions about their health and consequently exercise more control over their lives [8–10].

In spite of evidence of the worth of SDM, few health professionals have implemented shared decision making in routine clinical practice [11]. And in spite of many attempts to train health professionals to practise SDM, according to a Cochrane review of 39 studies of interventions to increase the use of SDM among HCPs, we still cannot say which interventions, if any, are effective [12]. However, the review suggested that interventions targeting both patients and physicians work better than those targeting one or the other. In other words, focusing on only one party is not enough to achieve widespread implementation of SDM. Some studies have begun to focus on the patient as a powerful agent for changing physician behaviour [13–15]. They have found evidence that patient expectations and demands can change physician behaviour, including their SDM behaviours. In one study, for example, when patients made explicit demands for anti-depression medication, their physician enacted more SDM behaviours and provided improved care for the patients [16]. The authors suggested that physicians only change paternalistic behaviours when a patient signals interest in SDM. Another study on patient participation in medical consultations found that 84% of active participation behaviours were initiated by patients rather than prompted by physicians [17].

Patients who are aware of SDM and knowledgeable about its benefits may thus be more likely to prompt physicians to adopt SDM behaviours. If patients are to be fully engaged in decision making, the public must also be trained in how to understand and evaluate risks, ask questions and express their values and preferences, because the public consists of potential patients and their relatives.

One way to inform the public about the benefits of SDM is through public libraries[18]. Public libraries are embedded in their communities and meet a range of information needs of diverse populations, including their needs for health information[19]. A US study found that health information was among the top five to ten topics of interest to library patrons, and that health requests accounted for 6% to 20% of their total reference requests [20,21]. The information role of libraries has expanded from providing purely text-based information to also providing web-based and electronic information, as well as live activities for community members. In Quebec in 2015, 96.2% of the population was served by public libraries and over 1.5 million Quebecers attended activities held at their public library [22]. There have been increasing calls for studies conducted in real-world settings with real-world constraints that reflect the practical realities of community and practice rather than in the highly controlled settings often found in efficacy studies. This has been described as an “important gap in the research literature” [23]. We considered public libraries to be an ideal setting for finding out whether our intervention was truly acceptable to its intended audience, i.e. potential patients.

A health issue that affects most members of the public is acute respiratory tract infections (ARTI). This issue is an appropriate vehicle for raising awareness about SDM, as whether to use antibiotics or not for ARTI is one of the most common decisions faced in primary care consultations. In addition, a) there is ample evidence about the importance of SDM for this decision [6], b) the implications of the treatment choices are not well known to the public, c) it could be of interest to individuals of all ages and conditions, d) it is a preference-sensitive decision [24], and e) overuse of antibiotics is a major public health problem [7].

With the goal of encouraging people to participate more actively in decisions about their health and scale up SDM so that more individuals can benefit from it, we investigated the feasibility, acceptability and impact of a program in public libraries to increase public awareness and knowledge about SDM and antibiotics use for ARTI.

## Materials and methods

### Study design

We carried out a feasibility study with a retrospective pre-post test design in a real-world setting. Primary outcomes were feasibility, acceptability and potential impact (limited efficacy testing) [23]. Public participant outcomes were measured retrospectively using a pre-post workshop questionnaire; facilitator outcomes using a post-test focus group and questionnaire.

### Participants

Participants were the general public and the facilitators. Eligible participants from the general public were 1) 12 years old or older (age limit for access to Quebec City Public libraries activities) and 2) exposed directly or indirectly to one of the workshop advertisements and interested enough to attend. The workshops were advertised using posters, radio announcements and radio interviews and by sending emails to public library users. Interested participants were invited to preregister.

Facilitators were 10 speakers, all physicians (six family physicians and four emergency physicians) from the Department of Family Medicine and Emergency Medicine at Université Laval, who presented the information, and a science communication specialist [25], who acted as an interpreter and facilitator.

### Intervention

#### Concept development

In partnership with the Quebec City public library network, a study proposal was co-developed and subsequently awarded a Canadian Institutes of Health Research (CIHR) dissemination grant (CIHR 143965). In this first phase, two meetings were held with the principal investigator (FL), the project coordinator (HR), two managers of the Quebec City Public Library Network (JG and MESH) and the SCS (VB). They discussed the format of the activity, the project timeline and the clinical topic to be presented at the workshop. The intention was to give the workshop free of charge, in different public libraries across the city, on various days (including the weekend) and at various times of the day (9 a.m., 11 a.m., 2 p.m., 7.30 p.m.) to maximize the number of participants and their diversity.

#### Workshop design and development

In the second phase, an executive committee that included the scientific communication specialist (VB), an information specialist and manager of the Quebec Public Library Network (MESH), the principal investigator (FL), who is an expert in SDM and knowledge translation, and the project coordinator (HR) met four times over two months to co-design a 1.5-hour interactive workshop. Participating physicians contributed to finalizing the format of the workshops, each of which was to be led by the scientific communication specialist (VB) and one physician. Ten workshops, one per library, were planned, but as activities in libraries are generally scheduled at least six months in advance, only nine libraries were available by the time the project began. However, one of the libraries agreed to host the event twice. All 10 workshops addressed the same topic of SDM and antibiotics. Each workshop held in a meeting room at each participating public library was to be facilitated by the scientific communication specialist, along with either a family physician or an emergency physician. The workshop consisted of an interactive presentation (60 minutes) using a PowerPoint^TM^ slideshow (S1 File) (Microsoft, Redmond, CA, USA) followed by a question and answer session (30 minutes).

Prior to holding the workshops, three one-hour meetings were held with the executive committee, the scientific communication specialist and the physicians to review and discuss the content and format of the workshop. The final version was slightly adjusted according to their comments and recommendations.

The library workshops would proceed as follows:

a) Participants (public) receive a series of documents including the decision aid, health information about antibiotics and resistance to antibiotics, and an evaluation form (S2 File). b) They are invited to complete the evaluation form and hand it in at the end, but to keep the information and the decision aid. Participants were told that by completing the evaluation form they consent to participate in the study. The evaluation form assesses participants’ satisfaction level, knowledge level after and before the workshop, opinions, and suggestions for improvement of the workshop. c) The scientific communication specialist and the accompanying physicians present workshop content, divided into five main sections: 1) introduction to ARTI; 2) introduction to antibiotics; 3) introduction to SDM; 4) presentation of a training program on SDM and antibiotics for ARTI entitled DECISION+ (including a decision aid); and 5) a simulated clinical encounter role-played by the communication specialist (as a simulated patient) and the participating physician to show participants how a physician and a patient can engage in SDM about the use of antibiotics for treating ARTI. During this presentation, the scientific communication specialist asks the physician questions in lay terms and helps clarify any statements that seemed misunderstood by the public. They also present the decision aid and explain what it is for and how it is used. d) At the end, the scientific communication specialist moderates a question-answer session with the public, explaining the answers in lay language if necessary.

All workshops were held from October 12 to November 30 2016.

### Data sources and measurement

Data were collected on participants' sociodemographic characteristics such as age (divided into six ranges), sex and occupation. The primary outcomes were related to feasibility. Feasibility components included participation rate, consent rate, acceptability (participants’ satisfaction, comprehension, etc.) and potential impact, e.g. on their knowledge about SDM and antibiotics. To enrich our results, qualitative data was also collected from three different sources: 1) Open-ended questions in the evaluation form of workshop (public) participants; 2) Verbatim of the post-workshop focus group among facilitators; and 3) Open-ended questions in the evaluation form (S3 File) completed by facilitators. This one-hour post-workshop focus group was held with the facilitators (the physicians, the science communication specialist and a library manager) after all the workshops. The focus group consisted of: 1) debriefing on the workshops, 2) filling out an evaluation form adapted from the one for general public participants on their satisfaction, knowledge gain, opinions and suggestions for improvements, and 3) a 20-minute open discussion.

Participation rate was the proportion of potential participants who pre-registered and subsequently participated in the workshops. Consent rate was the proportion of participants who agreed to fill in the evaluation form. We did not collect data on reasons for refusal to fill in the evaluation. Acceptability was measured using 12 closed-ended questions about participants' opinions on the workshop. Three questions were on the workshop quality and relevance, four were on the structure of the activity, three were on the workshop facilitation and two were on whether the workshop met their expectations and whether they would recommend it to others. Answers were chosen from a four-point Likert scale (ranging from ‘1 = totally disagree’ to ‘4 = totally agree’). Participants indicated their overall satisfaction with the workshop using a continuous ten-point scale where 0 corresponded to unsatisfied, 5 moderately satisfied and 10 fully satisfied. Open-ended questions invited participants to add suggestions, improvements and recommendations about topics for further workshops that might interest them. To assess the potential impact of the intervention, self-reported changes in SDM knowledge and in antibiotics knowledge were measured. Self-perceived knowledge levels about antibiotics and SDM pre- and post-workshop, assessed retrospectively, were measured using a continuous scale from 0 to 10. The comparison was made only among participants who had evaluated their knowledge levels both before and after the workshop. This retrospective pre-post method (as opposed to the traditional pre and post method) was used to eliminate response shift bias. Response shift bias, i.e. when a participant uses a different frame of understanding about a question between the pre and post periods, can mask program effectiveness. The retrospective method is also less burdensome for participants and precludes pretest sensitivity [26–28]. The face and content validity of these impact measures were established earlier through studies using similar questionnaires for assessing workshops designed for clinicians and adapted for patients. We have used these measures with success in numerous studies[29,30].

The facilitators’ evaluation form contained similar open-ended questions to those in the public participants’ evaluation form but adapted to their perspective (S3 File). Two 4-point scales (1 = totally disagree to 4 = totally agree) were added regarding the duration of the workshop and whether it was conducive to fruitful dialogue. Discussions were audio-recorded and transcribed.

As this study was supported by a dissemination grant and the workshops were part of the library system’s regular programming, the Research Ethics Board (REB) of the CHU de Québec judged that ethics approval was not required.

### Data analysis

Participants’ sociodemographic characteristics, opinions, perceptions, and satisfaction levels about the workshops were collected using descriptive statistics. To analyze opinions on the workshop, ‘totally disagree’ and ‘disagree’ were combined, as were ‘agree’ and ‘totally agree’, because too few people chose the category ‘totally disagree’. Normality of quantitative variables (such as satisfaction level, self-reported knowledge about antibiotics and SDM) was checked. The mean and the standard deviation, the median and interquartile interval, the minimum and the maximum were used to describe such variables. Paired T-test was used [31,32] to compare self-reported pre-post knowledge levels. Potential modifying effect of sociodemographic characteristics in the self-reported knowledge gains was checked by using T-test and ANOVA [32,33]. Sensitivity analyses were performed using nonparametric Kruskal Wallis [34] test to reinforce or not the results obtained by ANOVA [32]. The statistical threshold was set at 5%. Also a 95% confidence interval (CI) of the mean of self-reported knowledge gain was computed. If the 95% CI did not include 0, we considered the knowledge gain significant [35]. Sensitivity analyses were performed to assess the influence of missing data (n = 9) on the results. Analysis was performed using the software SAS 9.4.

Analysis of qualitative data on feasibility was performed using inductive and deductive reasoning according to a framework of feasibility components. The framework is based on eight main areas of focus in feasibility studies proposed by Bowen et al. [36]. Main themes were derived from quotes identified in the answers and verbatim transcripts of the three sources mentioned above. Then these main themes were classified within one of the eight components of feasibility.

## Results

Nine out of 24 public libraries participated in the project. From October 12 to November 30, 2016, all 10 planned workshops were held. Each workshop was facilitated by one of the 10 physicians assisted by the science communication specialist.

**Fig 1:**
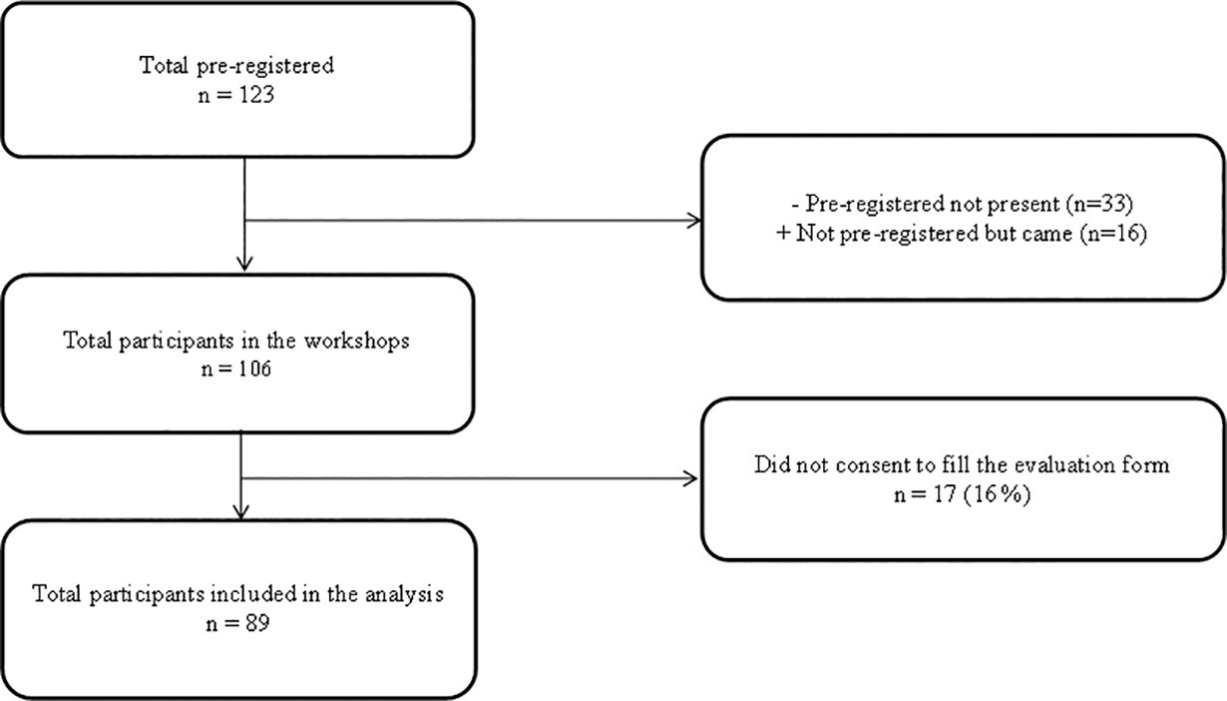
Flow chart of public participants.

One-hundred twenty-three (123) people were pre-registered. Thirty-three (27%) pre-registered people did not attend. Sixteen did not pre-register but attended. No-one was excluded from the workshop even if they did not pre-register. A total of 106 participants were finally recruited. Seventeen of the participants (16%) declined to complete the evaluation form (Fig 1). See Table 1 for a description of the 89 participants who completed the study.

**Table 1:** Public participants’ sociodemographic characteristics.

Sociodemographic variables	Number of participants (n = 89)	Percentage (%)
**Sex**		
Women	69	77.6
Men	10	11.2
Missing data	10	11.2
**Age (years)**		
16–30	9	10.1
31–45	16	18.0
46–60	17	19.1
61–75	30	33.7
76+	6	6.7
Missing data	11	12.4
**Occupation**		
Retired	42	47.2
Full-time employed	14	15.7
Students	11	12.3
Part time employed	3	3.4
Unemployed	2	2.3
Missing data	17	19.1

### Participant characteristics

Participants (general public) were mostly women (77%) over 45 years old (59.5%). Most of the participants were retired (47.2%). Those who were still working represented 19.0% of the total and 12.3% were students (Table 1).

Participants (facilitators) in the post-workshop focus group were five physicians, the principal investigator, one science communication specialist and a library information specialist. They were between 31 and 60 years old.

### Feasibility: Acceptability

#### Quantitative

Out of 123 people who preregistered for the workshops, 106 attended. Thirty-three preregistered participants did not attend for unknown reasons, while several people (n = 16) who had not registered did attend. The participation rate was 86.1%. The consent rate was 84.0%, i.e. out of the 106 attendees (range: 4 to 19 attendees per workshop), 89 participants accepted to complete the evaluation form and were included in the analysis (Fig 1).

Most public participants evaluated the activity content as relevant (94.4%), accessible to a lay audience (93.2%) and thought the information presented was clear (98.9%). On average, 95.5% of participants agreed or totally agreed with the quality and relevance of the workshop. They felt the stated objectives were met (92.1%) and that the information handed out was useful (94.4%). The majority of participants felt the workshop met their expectations (93.3%) and 94.4% would recommend the activity to others (Table 2). The item that scored lowest was “active participation” (86.5%). Satisfaction level did not fit normal distribution. The level of satisfaction reported by the participants varied between 0.5 and 10. The median satisfaction level was 9.0 (interquartile interval–IQR = 8.7–10) out of 10. Seventy-five per cent (75%) of participants reported a level of satisfaction greater than 8.7/10. Among these participants, 25% stated they were fully satisfied and their expectations were fully met (10/10).

**Table 2:** Public participants’ opinions on the activity (n = 89).

Items*	Participants’ opinions*	
	Disagree/Totally disagree n (%)	Agree/Totally agree n (%)
**Quality and relevance**		
Content accessible to a lay audience	4 (4.5)	83 (93.2)
Clear information	0	88 (98.9)
Relevant content	1 (1.1)	84 (94.4)
**Structure of activity**		
Goals were achieved	1 (1.1)	84 (94.4)
Time was sufficient	2 (2.3)	82 (92.1)
I participated actively	6 (6.7)	77 (86.5)
Documentation is useful	2 (2.3)	84 (94.4)
**Facilitation**		
Atmosphere conducive to conversation	2 (2.3)	86 (96.6)
Good complementarity between facilitators	2 (2.3)	85 (95.5)
Facilitators answered questions in a clear and practical way	1 (1.1)	84 (94.4)
**Globally**		
Workshop met my expectations	3 (3.3)	83 (93.3)
I would recommend this activity	3 (3.3)	84 (94.4)

* Missing values explain differences in the number of participants for each item.

#### Qualitative

Twenty-six main themes were identified from the three qualitative sources and were classified under eight key components of feasibility according to whether participants’ comments were linked to an aspect that they appreciated (+) or they thought could be improved or modified (-) (S1 Table). Briefly, participants and speakers most appreciated a) that the workshop was intended for the general public; b) the subject of the workshop (shared decision making and antibiotics); c) the discussion between facilitators; d) that the language was accessible to a lay audience; e) the clarity of the information; f) the involvement of experts (e.g. physicians); g) the question period; h) interactivity of the workshop; i) the documentation provided; and j) the format of the workshop. Participants (public and facilitators) suggested the workshops could be better publicized; could provide more time for questions; could be longer; could be adapted to other contexts; could be adapted to a video format; and could be expanded to reach other types of participants. Facilitators in addition suggested that future workshops should include payment for participating speakers; should be closer to their home or workplace; and that they involve patients more as speakers or to report testimonials. They reported total hours devoted to the project ranging from 8 to 100 (median = 10 hours).

Feasibility: Potential impact

Participants reported that they were fairly knowledgeable about antibiotics before the workshop (mean = 6/10) and more so afterwards (8.4/10), a significant (*P <* .*001*) increase in knowledge of 2.4 (95% CI: 2.0–2.8). SDM was less known to participants before the workshop (4.7/10). The workshop raised significantly (*P <* .*001*) their average level of knowledge by 4.0 (95% CI: 3.4–4.5) (Table 3).

**Table 3:** Self-reported knowledge gain about antibiotics and SDM (N = 80*).

	AB_after_	AB_before_	AB_gain_	p	95% IC gain
Mean (Sd)	8.4 (1.1)	6.0 (2.3)	+2.4 (1.8)	<0.001^a^	2.0–2.8
Min–Max	5–10	1–10	0–7	-	-
	SDM_after_	SDM_before_	SDM_gain_	p	95% IC gain
Mean (Sd)	8.7 (1.2)	4.7 (1.2)	+4.0 (2.4)	<0.001^a^	3.4–4.5
Min–Max	3–10	0–10	0–9	-	-

AB = antibiotics; after = after the workshop; before = before the workshop; Min = Minimum; Max = Maximum; Sd = standard deviation

a = Paired T–test

*Missing values (9) are about participants who did not answer the questions about self-reported knowledge levels.

**Fig 2:**
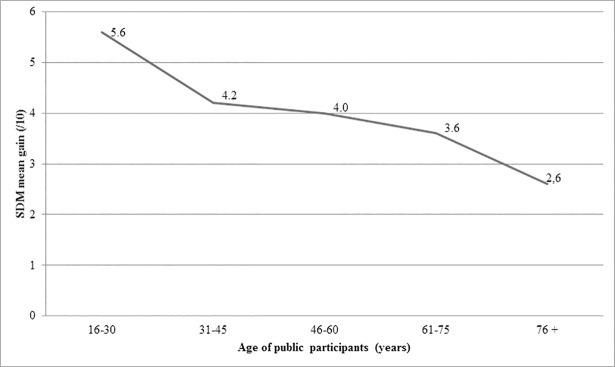
SDM knowledge gain by age of public participants.

Average knowledge gain about SDM was higher than about antibiotics (*P <* .*001*). The average knowledge gain did not vary according to sex (*P*_*AB*_ *=* .*187*; *P*_*SDM*_ *=* .*507*). Age did not seem to affect knowledge increase on either topic. However, knowledge gain about SDM tended to be less as participants’ age increased (*P =* .*239*) (Fig 2). The increase of knowledge about antibiotics and SDM did not vary with employment status (*P =* .*493* and *P =* .*052* respectively).

## Discussion

This study evaluated whether a series of public workshops held in a public library network to raise awareness among the general public about SDM and about antibiotic use for ARTI was feasible, acceptable and had an impact on participants’ awareness of SDM and antibiotics. The workshop content was co-designed by a large group of diverse stakeholders and experts and was considered feasible and acceptable by participants as well as by facilitators. It also showed that the intervention on a larger scale could have an impact on knowledge. Our results lead us to make three main observations.

First, we succeeded in creating a successful partnership with a key community player in knowledge dissemination: public libraries. Most participants in the study were women (77%), while 51% of Quebec city’s overall population are female Higher attendance of women could reflect the fact that women often seem to be more attentive to their health than men but also that they are more likely to use public libraries. Although the workshops took place during weekends and evenings to give time to younger families and workers who wanted to participate, most of the participants were retired, perhaps because they have more free time and also tend to have more health concerns [37,38] but also perhaps because they too are more likely to spend time in public libraries. These results suggest that public libraries may be especially appropriate for disseminating research results to this specific segment of the population.

Second, participants were very positive about the workshops overall. The communication of information was highly rated, probably due to the participation of the scientific communication specialist who is an expert in popularizing scientific results. Participants also appreciated the presence of a physician to answer their concerns directly. This could be due to the difficulty of accessing physicians in the Quebec region. It could also demonstrate the credibility that the profession brings to public discussion of health issues [39]. Indeed, the delivery of information about SDM by physicians may also have reassured patients that engaging in SDM in future medical consultations is likely to be acceptable to their physicians. On the other hand, participants gave their lowest scores for their own active participation in the workshop. The workshop format could have been a barrier to discussion since the question session was restricted to the end of the workshop. Allowing questions throughout the workshop could facilitate more participation. As participants suggested, adding time might increase participation, as would more interactive activities such as testimonials and role play.

Finally, the general public increased their knowledge about both SDM and antibiotics. They started at a lower level about SDM than about antibiotics and learned more about SDM than about antibiotics. While overuse of antibiotics has been covered extensively in the mainstream media [40], SDM has not been widely adopted in current medical practice and lack of knowledge about SDM among all stakeholders, including patients, has been identified as a major barrier to its implementation[41]. In addition, SDM is a more recent and perhaps more complex notion to grasp than is antibiotics. As knowledge gain about both SDM and antibiotics was significant, we wanted to see if this was the prerogative of a particular social slice. No obvious relationship was detected, although there was an inverse dose-response trend between SDM knowledge increase and participants’ age (Fig 2). Although the trend was not significant, younger people seem to have gained more knowledge about SDM than older people, perhaps because the latter started with a higher level of knowledge (results not presented). This in turn could be explained by the fact that older people are higher consumers of care than younger people and may have more experience of SDM or a better idea of what SDM could be [42–44]. Another explanation of the lower knowledge gain among older people might be that they need the knowledge presented in a different form. These hypotheses require further exploration, as the results will affect the content and targeted participants of future SDM interventions aiming to raise awareness among the general public.

This feasibility study has limitations. Participants were older than the average Quebec City population and were thus not representative. No statistics on the characteristics of people who use libraries in Quebec City were available, and so it was impossible to determine if the studied population was representative of public library users, either. On the evaluation sheet, the question related to overall satisfaction on a scale of 10 (“Indicate with a vertical line on the following visual analogue scale your level of overall satisfaction with the workshop”) was not well understood by all the participants, including some clinicians. Communication channels used to advertise the program were limited and a future initiative would need to advertise it more broadly. Lastly, future initiatives should integrate patient representatives in the development of the workshops as well as in their facilitation.

## Conclusions

The aim of this study was to investigate the feasibility, acceptability and potential impact of a program to raise awareness of SDM and antibiotics use among the general public and ultimately contribute to the scale up and spread of SDM so that more individuals can benefit from it. The program consisted of a series of interactive public library events about the impact of SDM on the use of antibiotics to treat ARTI. Results showed the strategy is feasible and acceptable for raising awareness of SDM among the general public and we plan to improve it and offer the program to an even larger audience. This study offers a new approach to implementation of SDM about tests and treatments. The public we reached are all potential patients, parents, and grandparents of potential patients. While programs to foster SDM among health providers have not resulted in its widespread implementation, this project opens the door to a new way to implement SDM, this time through preparing the public and patients themselves to ask for it.

## Supporting information

S1 Table Qualitative analysis. Symbol + means “most appreciated”. Symbol–meaning “to be improved or to be modified”.
(PDF)

S1 File Powerpoint presentation during workshops. (PDF)

S2 File Public participants’ evaluation form. (PDF)

S3 File Facilitators’ evaluation form. (PDF)

S4 File STROBE checklist for items. (PDF)

S5 File Database participants. (XLSX)
