# Supplementary material for: Can patients be trained to expect shared decision making in clinical consultations? Feasibility study of a public library program to raise patient awareness
Source: PLoS One. 2018 Dec 12;13(12):e0208449. doi: 10.1371/journal.pone.0208449 (PMC6291239; doi:10.1371/journal.pone.0208449)
Supplement: S1 Table — Symbol + means “most appreciated”. Symbol–meaning “to be improved or to be modified”. (PDF) [file pone.0208449.s001.pdf]

Table: Qualitative analysis

			Source and number of quotes			
**Key component of feasibility**	**Main themes associated with the key component identified**	**+/-**	**Post-workshop discussion session**	**Facilitator evaluation**	**Public participant evaluation**	**Example of quote**
**Acceptability**	General public as the target	+	1			*"It's great to have an activity like this for the general public in a relaxed atmosphere"*
	Shared decision making and antibiotics as the subject	+			5	*"I learned about shared decision making"* *"The activity fits with the priorities of funding agencies and governmental organisations on controlling antibiotics use"*
	Role of speakers	+			4	*"The speakers spoke in a simple way, this was a good point"*
	Discussion between speakers	+	1	5	11	*"Interesting dialogue between the speakers"*
	Simplified terms	+			12	*"It was good that the language could be understood by ordinary people"*

	Clarity of the information	+			15	*"The information presented was very clear and easy to understand"*
	Involvement of experts	+		5	5	*"Presence of a physician"*<br><br>*"Presence of a specialist in scientific communication"*
	Location	+			1	*"Libraries are a good location, good way to use libraries"*
**Demand**	Advertising	-	4	4	3	*"Important to have a good plan for publicity and do more advertising"*
**Implement-ation**	Collaboration of partners	+	1			*"The activity needs the collaboration of all the actors; the public library, the trainers and the scientific communicator"*
	Availability of trainers	+	1			*"Need to have backup if a trainer cannot attend"*
**Practicability**	Balance of time between speakers	-			1	*"More time for the physician"*
	Time for questions	+		1	5	*"Good to have time for questions"*
		-		1	4	*"Allowing questions after the first part of the workshop to promote interactivity"*<br><br>*"More time for questions"*
	Evidence-based content	+			2	*"The content is based on evidence"*

	Interactivity	+		1	6	*"Good interactivity with the speakers"*
		-	1			*"The format must not be too interactive if we do not want to lose control"*
	Length of the workshop	-		1	1	*"Make the workshop longer"*
	Documentation	+			9	*"The documents given out were helpful"*
**Adaptation**	Payment for speakers	-	1			*"Trainers will need to be paid if you want to expand this because of the time it requires"*
	Distance for speakers	-	2			*"Trainers would prefer to train in a library in their own community, where they live"*
	Format	+	4			*"Do not need to be adapted as a web tutorial"*
		-	3		1	*"The activity could be transferred to a digital format or video to be presented on screens in the waiting rooms of medical clinics and more widely distributed"*
	Patient involvement	-	6	1		*"Include a patient in the activity who could share his/her experience of engaging in shared decision making in his encounters with a health professional"*
	Context	-	1		1	*"The activity could be adapted to different contexts, such as emergency medicine"*

**Integration**	Integration in the location of implementation	+	1			*"This activity fits well with the mission of public libraries"*
**Expansion**	Participants	-	4	1	2	*"To expand the activity to include other type of participants maybe more typical to those encountered in regular family practice"*<br>*"Present to other groups such as schools (high schools), policemen, fire fighters, social groups, community groups"*
Limited-efficacy testing	Knowledge	+	1			*"The program has led to a significant increase in the perception of knowledge gain"*
	Measurement	+	2			*"The retrospective pre-post test allows participants to have a better understanding of what they are being asked to assess"*

+ : Most appreciated

- : To be improved or to be modified
