# Supplementary material for: Can patients be trained to expect shared decision making in clinical consultations? Feasibility study of a public library program to raise patient awareness
Source: PLoS One. 2018 Dec 12;13(12):e0208449. doi: 10.1371/journal.pone.0208449 (PMC6291239; doi:10.1371/journal.pone.0208449)
Supplement: S1 File — (PDF) [file pone.0208449.s002.pdf]

Public lectures

My health, my decisions

ANTIBIOTICS, A DECISION TO SHARE

UNIVERSITÉ LAVAL

Canada Research Chair in Shared Decision Making and Knowledge Translation

Chaire de recherche du Canada en décision partagée et application des connaissances

Centre intégré universitaire de santé et de services sociaux de la Capitale-Nationale Québec

UNITÉ DE SOUTIEN SRAP | QUÉBEC

Institut universitaire de première ligne en santé et services sociaux

UNIVERSITÉ LAVAL

Faculté de médecine

Département de médecine familiale et de médecine d'urgence

CHU de Québec

CIHR IRSC

L'Institut Canadien de Québec

VILLE DE QUÉBEC

Bibliothèque de Québec

Découvrir. Se divertir. Rêver.

1

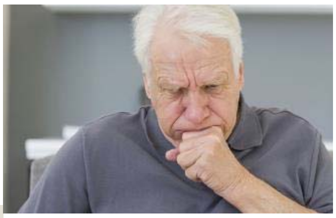

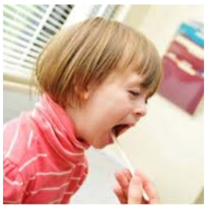

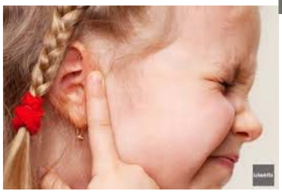

Introduction

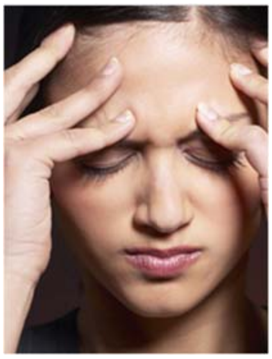

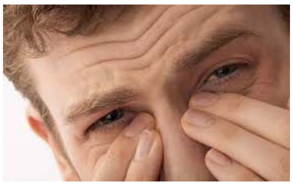

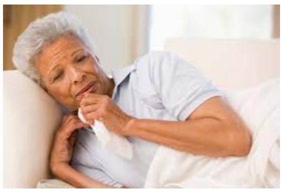

2

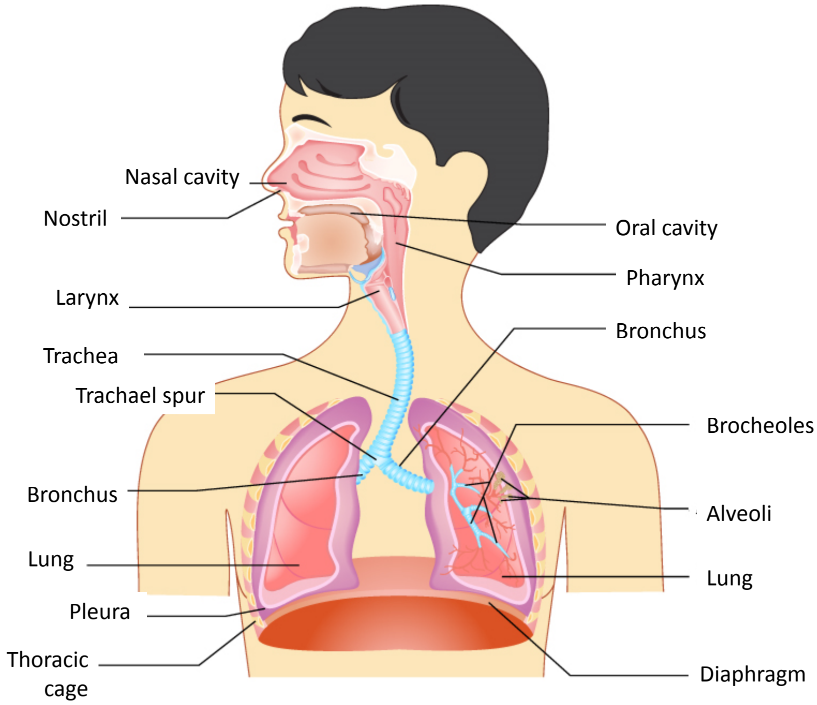

3

What causes respiratory tract infections?

- Bronchitis is nearly always caused by a virus (more than **9/10**)
- Pharyngitis (sore throat) is often caused by a virus (**8/10**)
- Acute otitis media (ear infection) is often caused by a bacteria (**9/10**)

4

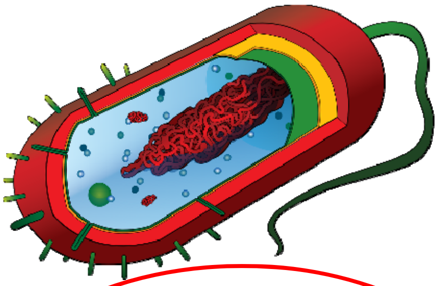

Bacteria
- ➢ Has a cell
- ➢ Is sensitive to antibiotics

Eg: streptococcus, pneumococcus

Virus
- ➢ No cells (1000 times smaller than bacteria!)
- ➢ Not sensitive to antibiotics

Eg: rhinovirus, adenovirus, coronavirus

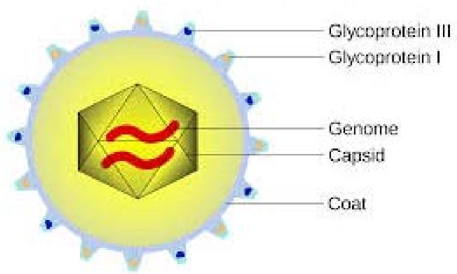

When should I see the doctor?

- *Info santé* helpline: 811
- *Santé mieux-être* website: sante.gouv.qc.ca/en
- *From Tiny Tot to Toddler*
  - Fever (rectal ≥ 38.1°) in babies under 6 mths
  - Fever for 3 days

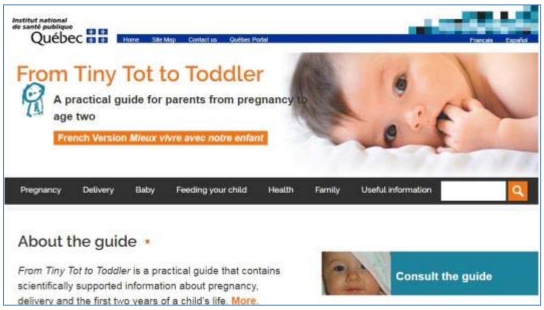

Eardrums and otitis

- **If it's painful:**

Inflammation and fluid	Inflammation but no fluid	No inflammation, no fluid
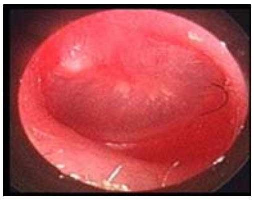	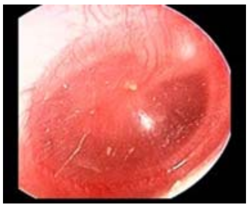	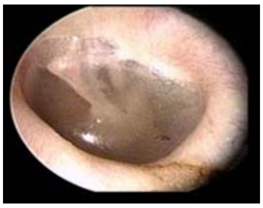
99% chance of bacteria	60% chance of bacteria	3% chance of bacteria

- **If it's not painful: the chances are different!**

7

ACUTE RHINOSINUSITIS

To differentiate patients with an ACUTE RHINOSINUSITIS due to a bacteria from those whose ACUTE RHINOSINUSITIS is due to a virus

STEP 1

Tick all the key symptoms and signs identified in your patient with symptoms of rhinosinusitis

INITIAL QUESTION
Duration of symptoms
- ☐ < 10 days ☐ ≥ 10 days

ADDITIONAL QUESTIONS
- ☐ Double sickening (worsening after improving)
- ☐ Colored nasal discharge
- ☐ Facial/sinus pain
- ☐ Maxillary tooth pain
- ☐ No response to decongestants

ADDITIONAL SIGNS
- ☐ Purulent discharge in nasal cavity (middle meatus) and/or throat
- ☐ Sinus pain on **one side**
- ☐ Abnormal transillumination (**one side**)

ALERTS

- Persistent high fever
- Severely ill
- Orbital swelling or erythema
- Diplopia, proptosis or other neurologic signs

STEP 2

Encircle the clinical probability (%) of a bacterial acute rhinosinusitis according to signs and symptoms of patients assuming a prevalence of 15%

Additional symptoms/signs	<10 days	>10 days*
4+	30%	95%
3	15%	75%
2	5%	50%
1	2%	25%
0	1%	5%

*Adults 7-10 days; children 10-14days

Whatever kind of bug it is (virus or bacteria), on average...

- **With bronchitis**, the cough will disappear in **24 days**
- **With otitis (ear)**, the pain will disappear in **3 days**
- **With pharyngitis (throat)**, the pain will disappear in **5 days**
- **With sinusitis**, the congestion will disappear in **10 days**

Supportive treatment (e.g. Tylenol) can help

Antibiotics

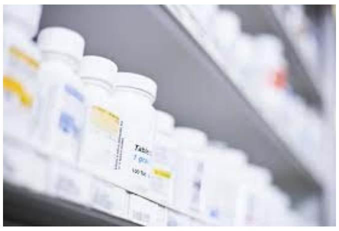

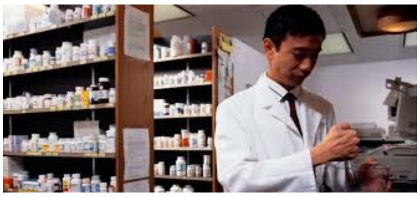

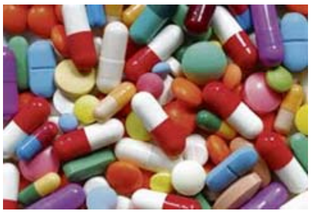

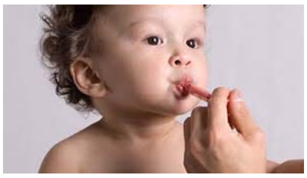

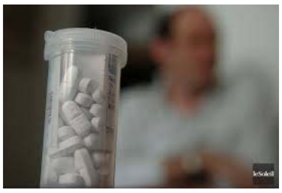

A leap forward

1920: In Quebec, one in three people died of an infectious disease (germ-caused)

2016: less than 1% of deaths are due to germs

1920: A quarter of newborns died before they were year old

2016: about 1 out of 250 die.

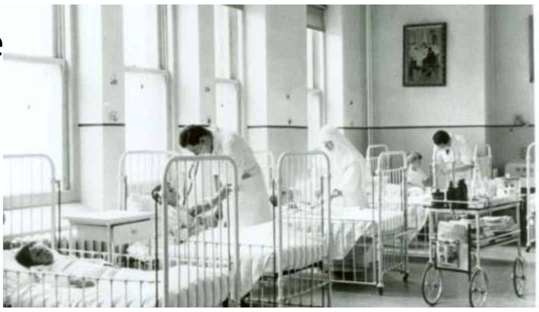

Pediatric ward, Hôtel Dieu de Québec, ca. 1930 (Archives du monastère des Augustines)

What made the difference? Hygiene, vaccines et antibiotics!

11

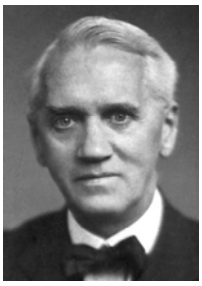

Then and now

1928: Alexander Fleming discovered penicillin

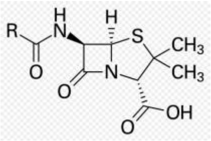

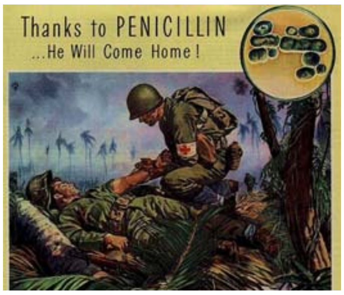

1944: streptomycin (1st antibiotic that worked against tuberculosis)

1950-80: erythromycin, vancomycin, fluoroquinolones…

Since then, different formats but hardly any new antibiotics

About 30 antibiotics are commonly used in Canada

12

What are antibiotics used for?

- To fight **infections caused by bacteria,** for example:

  Syphilis, gonorrhea, cholera, plague, E. coli diarrhea, C. difficile, flesh-eating bacteria, bacterial urinary infection, bacterial meningitis, streptococcal pneumonia … and bacterial respiratory infections
- Main antibiotics used against bacterial upper respiratory tract infections:

  Amoxicillin, clarithromycin, azithromycin, erythromycin, penicillin
- Antibiotics used against viral infections: **NONE**!

13

How do antibiotics work?

Inhibit cell wall synthesis:
Beta-lactams, glycopeptides, fosfomycin

Act on plasma membrane:
Polymixins

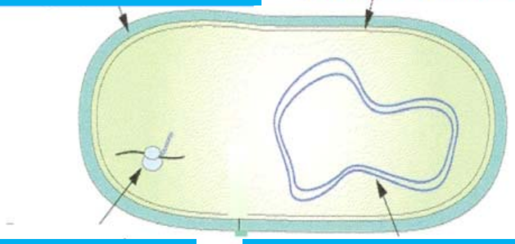

Inhibit protein synthesis:
Tetracyclines, aminoglycosides, chloramphenicol, macrolides, fucidic acid, linezolid

Inhibit nucleic acid synthesis or function:
Quinolones, sulfonamides, rifampicin, 5-nitroimidazoles, nitrofurantoin

Targets of antibiotic action

Only on bacteria!

14

Risks and side-effects

- Severe allergies to antibiotics: 4-15/100,000

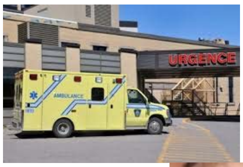

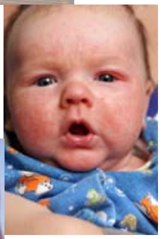

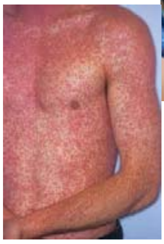

- Side-effects (vomiting, diarrhea, rashes…): 5/100
- Viruses and antibiotics don't always get along: this rash was from using ampicillin for mononucleosis

15

Over-prescribed medications

- **More than 2/3** of acute respiratory infections are caused by viruses.
- Yet 2/3 of these infections are treated with antibiotics!
- **One of three Canadians** was prescribed an antibiotic for an acute respiratory infection last year.
- Canadians take **250 tons** of antibiotics per year.

16

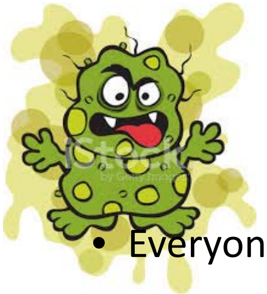

Why?

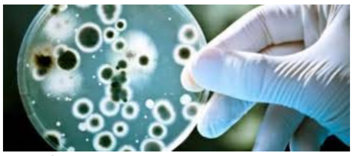

- Everyone is **afraid** of germs, including doctors!
- People think that **the only thing** that works is … antibiotics!
- Doctors and patients tend to overestimate the benefits of antibiotics and underestimate the risks

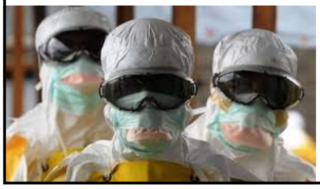

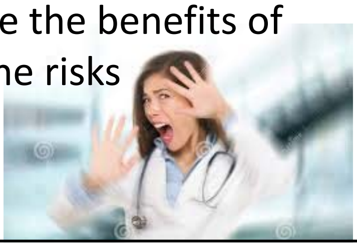

What is antibiotic resistance?

- Bacteria become resistant to antibiotics, so the antibiotics can no longer kill them.
- People transmit this resistance to their descendants.
- A natural adaptation process, but magnified by bad use of antibiotics.
- Resistance can be transmitted from one bacteria to another, **even when they're not of the same type**!

18

Antibiotic resistance: a very serious problem

Tens of thousands of deaths per year!

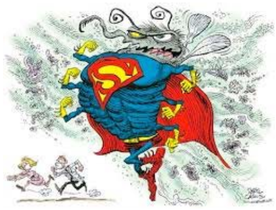

More and more bacteria are resistant:
the tuberculosis bacillus, staphylococcus…

Soon there may be no treatment against gonorrhea

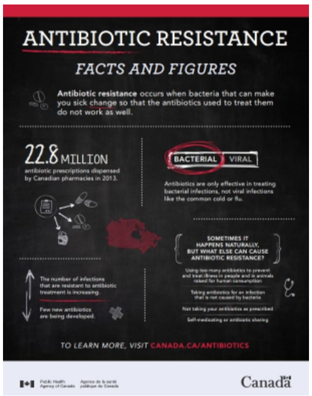

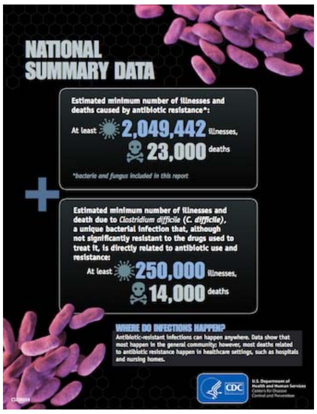

What can I do?

I can be responsible about taking antibiotics

<u>by only using them when needed and as prescribed</u>

We're all part of this!

20

If 100 people with an acute respiratory infection

… didn't take antibiotics

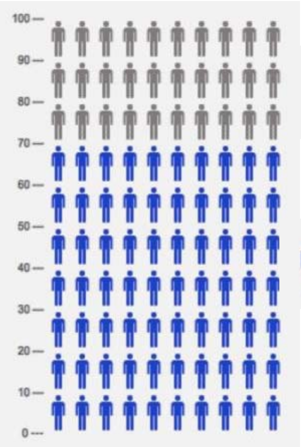

30 would still have symptoms

70 would be better

… did take antibiotics

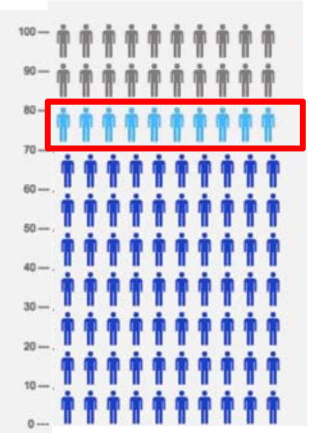

20 would still have symptoms

10 would have got better faster with antibiotics

70 would have got better without the antibiotics

21

If 100 people with an acute respiratory infection take antibiotics

90 are taking it for nothing!

20 are still sick after the average recovery time

\+

70 would have recovered without antibiotics

The other 10 people get better a little faster (a few hours or days) than if they hadn't taken antibiotics

22

Out of 100 people with an acute respiratory infection

…and who don't take antibiotics

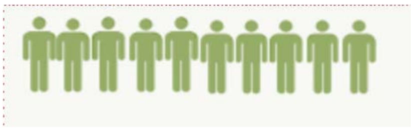

10 would have additional symptoms because of their illness (diarrhoea, stomach ache, vomiting…)

… and who do take antibiotics

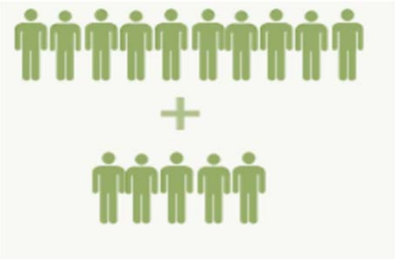

10 would have additional symptoms because of their illness (diarrhoea, stomach ache, vomiting…)

+

5 would have had side effects from the antibiotics

23

Shared decisions

Chaire de recherche du Canada
en décision partagée et application des connaissances

UNIVERSITÉ LAVAL

UNIVERSITÉ LAVAL

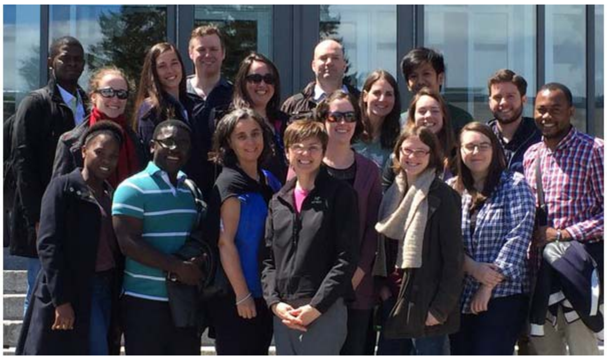

CHU de Québec

24

Five questions to ask

1) What is my problem?
2) What are my options?
3) What are the risks?
4) What will happen if I do nothing?
5) Am I clear about what's important to me?

Which do you prefer?

Options	Benefits<br>(why to choose this)	Risks<br>(why not to choose this)
**#1**<br>***Take an antibiotic***	• Slightly increases chances of relieving symptoms faster. + + + + +<br>• ……………….. + + + + +	• Taking antibiotics for several days. + + + + +<br>• Side effects (diarrhea, stomach ache, allergic skin reactions). + + + + +<br>• ………………………… + + + + +
**#2**<br>***Don't take an antibiotic***	• Heal naturally + + + + +<br>• Not exposed unnecessarily to antibiotics + + + + +<br>• …….. + + + + +	• Slightly reduces chances of relieving symptoms faster. + + + + +<br>• ………. + + + + +

Thanks for coming!

Public lectures

My health, my decisions

Canada Research Chair in Shared Decision Making and Knowledge Translation
Chaire de recherche du Canada en décision partagée et application des connaissances

Université Laval
Faculté de médecine
Département de médecine familiale et de médecine d'urgence

27
