# Supplementary material for: Can patients be trained to expect shared decision making in clinical consultations? Feasibility study of a public library program to raise patient awareness
Source: PLoS One. 2018 Dec 12;13(12):e0208449. doi: 10.1371/journal.pone.0208449 (PMC6291239; doi:10.1371/journal.pone.0208449)
Supplement: S2 File — (PDF) [file pone.0208449.s003.pdf]

UNIVERSITÉ LAVAL — Chaire de recherche du Canada en décision partagée et application des connaissances

Fiche d’évaluation de l’atelier « Ma santé c’est mon choix : Les antibiotiques, une décision partagée ».

Endroit (Bibliothèque) :<br><br>Date et heure (ex. : mercredi 12 octobre 2016; 14h):

À l’aide de l’échelle suivante, cochez la case qui correspond le mieux à votre opinion	**Pas du tout d’accord**	**Peu en accord**	**Plutôt d’accord**	**Tout à fait d’accord**
**Qualité et pertinence**				
1. Le contenu était adapté pour moi				
2. L’information présentée était claire				
3. Le contenu était pertinent				
**Structure de l’activité**				
4. L’atelier a permis d’atteindre les objectifs annoncés				
5. Le temps alloué aux différents points était suffisant				
6. J’ai pu participer de façon active				
7. La documentation remise me sera utile				
**Animation**				
8. Les animateurs ont su créer une atmosphère propice à l’échange				
9. Il y avait une bonne complémentarité entre les animateurs				
10. Les animateurs ont répondu aux questions de manière claire et pratique				
**Dans l’ensemble**				
11. L’atelier a répondu à mes attentes				
12. Je recommanderais cette activité				

Identifiez deux caractéristiques que vous avez particulièrement appréciées dans cet atelier :

1. ______________________________

2. ______________________________

Écrivez une ou deux suggestions pour améliorer cet atelier

1. ______________________________

2. ______________________________

Indiquer par un trait vertical sur l’échelle visuelle analogue suivante, votre niveau de satisfaction globale vis-à-vis de <u>l’atelier « Ma santé c’est mon choix : Les antibiotiques, une décision partagée»</u>

Très insatisfait — Très satisfait

0 |——————————————————| 10

VOIR VERSO

Université Laval — Chaire de recherche du Canada en décision partagée et application des connaissances

Comment évalueriez-vous votre niveau de connaissance sur les antibiotiques **avant** cet atelier?

0--------1--------2--------3--------4--------5--------6--------7--------8--------9--------10
Extrêmement faible — Modéré — Extrêmement élevé

Comment évalueriez-vous votre niveau de connaissance sur les antibiotiques **après** cet atelier?

0--------1--------2--------3--------4--------5--------6--------7--------8--------9--------10
Extrêmement faible — Modéré — Extrêmement élevé

Comment évalueriez-vous votre niveau de connaissance de la prise de décision partagée **avant** cet atelier?

0--------1--------2--------3--------4--------5--------6--------7--------8--------9--------10
Extrêmement faible — Modéré — Extrêmement élevé

Comment évalueriez-vous votre niveau de connaissance de la prise de décision partagée **après** cet atelier?

0--------1--------2--------3--------4--------5--------6--------7--------8--------9--------10
Extrêmement faible — Modéré — Extrêmement élevé

Autres sujets que vous aimeriez voir abordés par les médecins :

____

Autres commentaires et suggestions

____

Informez-moi des futures activités de « Ma santé, c'est mon choix ». **Encerclez :** OUI NON

Courriel : ____

ENCERCLEZ : Je suis : UNE FEMME UN HOMME

ENCERCLEZ : Quel âge avez-vous? 0-15 16-30 31-45 46-60 61-75 76 et plus

Quelle est votre occupation (étudiant, retraité, employé à temps complet, employé à temps partiel, sans emploi)? : ________________________ Autre :__________________

Merci d'avoir complété cette fiche d'évaluation.
