# Supplementary material for: Can patients be trained to expect shared decision making in clinical consultations? Feasibility study of a public library program to raise patient awareness
Source: PLoS One. 2018 Dec 12;13(12):e0208449. doi: 10.1371/journal.pone.0208449 (PMC6291239; doi:10.1371/journal.pone.0208449)
Supplement: S3 File — (PDF) [file pone.0208449.s004.pdf]

Chaire de recherche du Canada en décision partagée et application des connaissances

Evaluation form for debriefing on the project "My health, my choice: antibiotics, a shared decision"

Place: Hôpital Saint-François d'Assise, room D6-725

Date and time: Thursday April 6th, 2017; 1 pm to 3 pm

Check the box that best matches your opinion	Totally Disagree	Disagree	Agree	Totally Agree
**Quality and relevance**				
1. The content was understandable to the general public				
2. The information presented was clear for the general public				
3. The content was relevant for the general public				
**Activity format**				
4. Workshop length was appropriate				
5. The workshop achieved the advertised objectives (see Objectives)				
6. There was enough time for each point				
7. I was able to participate actively				
8. The documents given out would be useful for the general public				
**Facilitation**				
9. The atmosphere was conducive to discussion				
10. The facilitators complemented each other				
11. The questions for the public were relevant				
**In general**				
12. The workshop met my expectations				
13. The workshop format was conducive to discussion				
14. I would recommend this activity to my patients/friends/ family/clients				

Write down two things you particularly liked about the workshop:

1. ___

2. ___

Write down one or two suggestions for improving the workshop

1. ___

2. ___

Draw a vertical line on this scale to mark your overall level of satisfaction with the workshop "My health, my choice: antibiotics, a shared decision"

Very dissatisfied — Very satisfied

0 |———————————————| 10

Please answer the following questions about whether this project increased your knowledge on issues relating to your practice:

How would you rate your level of knowledge about antibiotics **before** your participation in this project?

0--------1--------2--------3--------4--------5--------6--------7--------8--------9--------10
Very poor Average Excellent

How would you rate your level of knowledge about antibiotics **after** your participation in this project?

0--------1--------2--------3--------4--------5--------6--------7--------8--------9--------10
Very poor Average Excellent

How would you rate your level of knowledge about shared decision making **before** your participation in this project?

0--------1--------2--------3--------4--------5--------6--------7--------8--------9--------10
Very poor Average Excellent

How would you rate your level of knowledge about shared decision making **after** your participation in this project?

0--------1--------2--------3--------4--------5--------6--------7--------8--------9--------10
Very poor Average Excellent

Other subjects you would like to see addressed:

Suggestions for improving the participation of the general public (attendance and participation within the workshop):

____
____
____
____

Other comments and suggestions:

____
____
____
____
____
____
____

Approximately how much time did you invest in this project (including preparation meetings, preparing for the workshop, transportation, giving the workshops, debriefing and other)?

__________ hours

If you could, would you be interested in participating in another, similar project?

Circle: YES NO

Circle: I am: A WOMAN A MAN

Circle: How old are you? 0-15 16-30 31-45 46-60 61-75 76 or more

What is your role in this project (student, doctor, researcher, facilitator, other)?

____

*Other:*__________________________

Thank you for filling out this evaluation form.
