# Supplementary material for: Can patients be trained to expect shared decision making in clinical consultations? Feasibility study of a public library program to raise patient awareness
Source: PLoS One. 2018 Dec 12;13(12):e0208449. doi: 10.1371/journal.pone.0208449 (PMC6291239; doi:10.1371/journal.pone.0208449)
Supplement: S4 File — (PDF) [file pone.0208449.s005.pdf]

STROBE Statement—Checklist of items

	Item No	Recommendation	Pages
**Title and abstract**	1	(*a*) Indicate the study's design with a commonly used term in the title or the abstract	Title : P.1; Line 1-5
		(*b*) Provide in the abstract an informative and balanced summary of what was done and what was found	Abstract : P.3 – 4; Line 43 –72
**Introduction**			
Background/rationale	2	Explain the scientific background and rationale for the investigation being reported	P.5-7; Line 76 – 135
Objectives	3	State specific objectives, including any prespecified hypotheses	P.7; Line 136 – 139
**Methods**			
Study design	4	Present key elements of study design early in the paper	P.7-8;Line 141 –146
Setting	5	Describe the setting, locations, and relevant dates, including periods of recruitment, exposure, follow-up, and data collection	P.9; Line 162 – 168.<br>P.9; Line 176 – 184.<br>P.10; Line 189 – 196.
Participants	6	(*a*) Give the eligibility criteria, and the sources and methods of selection of participants	P.8; Line 147 –157
Variables	7	Clearly define all outcomes, exposures, predictors, potential confounders, and effect modifiers. Give diagnostic criteria, if applicable	P.10 – 11; Line 212 – 217
Data sources/ measurement	8	For each variable of interest, give sources of data and details of methods of assessment (measurement). Describe comparability of assessment methods if there is more than one group	P.11 –12; Line 228 – 254
Bias	9	Describe any efforts to address potential sources of bias	P.12; Line 245 – 249
Study size	10	Explain how the study size was arrived at	N/A
Quantitative variables	11	Explain how quantitative variables were handled in the analyses. If applicable, describe which groupings were chosen and why	P.13; Line 270 – 272
Statistical methods	12	(*a*) Describe all statistical methods, including those used to control for confounding	P.13; Line 272 – 278
		(*b*) Describe any methods used to examine subgroups and interactions	P.13; Line 273 – 274
		(*c*) Explain how missing data were addressed	P.14; Line 279 – 280
		(*d*) If applicable, describe analytical methods taking account of sampling strategy	N/A
		(*e*) Describe any sensitivity analyses	P.13; Line 273 – 274

**Results**			
Participants	13	(a) Report numbers of individuals at each stage of study—eg numbers potentially eligible, examined for eligibility, confirmed eligible, included in the study, completing follow-up, and analysed	P.14; Line 285 – 290
		(b) Give reasons for non-participation at each stage	P.11; Line 230 – 231
		(c) Consider use of a flow diagram	P.14; Line 291 – 296
Descriptive data	14	(a) Give characteristics of study participants (eg demographic, clinical, social) and information on exposures and potential confounders	P.14 –15; Line 291–299
		(b) Indicate number of participants with missing data for each variable of interest	P.14; Line 279 – 280
Outcome data	15	Report numbers of outcome events or summary measures	P.15 –16; Line 300 – 319<br>P.16 –17; Line 323 – 339<br>P.17–18; Line 340 – 355
Main results	16	(*a*) Give unadjusted estimates and, if applicable, confounder-adjusted estimates and their precision (eg, 95% confidence interval). Make clear which confounders were adjusted for and why they were included	N/A
		(*b*) Report category boundaries when continuous variables were categorized	N/A
		(*c*) If relevant, consider translating estimates of relative risk into absolute risk for a meaningful time period	N/A
Other analyses	17	Report other analyses done—eg analyses of subgroups and interactions, and sensitivity analyses	N/A
**Discussion**			
Key results	18	Summarise key results with reference to study objectives	P.16 – 19; Line 306-362
Limitations	19	Discuss limitations of the study, taking into account sources of potential bias or imprecision. Discuss both direction and magnitude of any potential bias	P.21 – 22; Line 415 – 425
Interpretation	20	Give a cautious overall interpretation of results considering objectives, limitations, multiplicity of analyses, results from similar studies, and other relevant evidence	P.19 – 21; Line 371 – 414
Generalisability	21	Discuss the generalisability (external validity) of the study results	P.22; Line 431 – 434
**Other information**			
Funding	22	Give the source of funding and the role of the funders for the present study and, if applicable, for the original study on which	P.23; Line 447 – 449

		the present article is based	

Note: An Explanation and Elaboration article discusses each checklist item and gives methodological background and published examples of transparent reporting. The STROBE checklist is best used in conjunction with this article (freely available on the Web sites of PLoS Medicine at http://www.plosmedicine.org/, Annals of Internal Medicine at http://www.annals.org/, and Epidemiology at http://www.epidem.com/). Information on the STROBE Initiative is available at www.strobe-statement.org.
